# Metagenomic Analysis of the Gastrointestinal Phageome and Incorporated Dysbiosis in Children with Persistent Diarrhea of Unknown Etiology in Vietnam

**DOI:** 10.3390/pathogens14100985

**Published:** 2025-09-29

**Authors:** Trong Khoa Dao, Thi Thanh Nga Pham, Hong Duong Nguyen, Quang Trung Dam, Thi Bich Thuy Phung, Thi Viet Ha Nguyen, Thi Quy Nguyen, Kim Chi Hoang, Thi Huyen Do

**Affiliations:** 1 Institute of Biology, Vietnam Academy of Science and Technology (VAST), 18-Hoang Quoc Viet Street, Ha Noi 10072, Vietnam; khoadt@ibt.ac.vn (T.K.D.); duongnguyen96uet@gmail.com (H.D.N.); quynhungcuong@yahoo.com (T.Q.N.); 2 Vietnam National Children’s Hospital, 879 La Thanh Street, Ha Noi 10000, Vietnam; phamthithanhnga1986@gmail.com (T.T.N.P.); thuyphung.nhp@gmail.com (T.B.T.P.); vietha@hmu.edu.vn (T.V.H.N.); 3 Hospital of Post and Telecommunications, 49 Tran Dien Street, Ha Noi 10000, Vietnam; damquangtrung1206@gmail.com; 4 Department of Pediatric, Hanoi Medical University, No 1 Ton That Tung Street, Ha Noi 11100, Vietnam; 5 Institute of Chemistry, Vietnam Academy of Science and Technology (VAST), 18-Hoang Quoc Viet Street, Ha Noi 10072, Vietnam; chihoangkim@gmail.com; 6 School of Biotechnology, Graduate University of Science and Technology, Vietnam Academy of Science and Technology (VAST), 18-Hoang Quoc Viet Street, Ha Noi 10072, Vietnam

**Keywords:** bacteriome, dysbiosis, persistent diarrhea of unknown etiology, phageome, phage–host interaction

## Abstract

Persistent diarrhea of unknown etiology in children under 2 years of age is a common problem and poses a major challenge for the health sector. However, knowledge of the composition and dysbiosis of the intestinal phageome, phage-associated bacteriome in the persistent diarrhea remains limited. In this study, a process for phage enrichment and metagenomic extraction was developed and applied to recover gut phage metagenomes from 30 healthy children and 30 children with persistent diarrhea for high-throughput sequencing. Taxonomic annotation using Kraken2 revealed that, besides *Norwalk virus*, *Primate bocaparvovirus 1* and *Human-associated gemykibivirus 2*, phage communities in the diarrhea group showed reduced diversity and contained sample-dependent phages targeting *Salmonella enterica*, *Enterobacter*, *Shigella flexneri*, *Clostridioides difficile*, *Pseudomonas aeruginosa*, *Streptococcus miti*, uropathogenic *Escherichia coli* and functioned balancing bacterial communities. Bacterial fraction in the metagenomic datasets reflected clear patterns of dysbiosis, including a severe deficiency of beneficial bacteria, an increase in Firmicutes, a marked decline in Actinobacteria, Bacteroidetes, Proteobacteria and sample-dependent enrichment of *Enterococcus*, *Escherichia* and *Acinetobacter* in diarrhea cases. This study, for the first time, investigated the dynamics of gut phageome, phage-associated bacteriome in children with persistent diarrhea of unknown causes in Vietnam, providing new insight for complementary treatment.

## 1. Introduction

Infectious diarrhea is a global health problem, particularly among infants and young children, and remains a leading cause of morbidity and mortality. According to a report by the World Health Organization and UNICEF, diarrhea causes nearly two million deaths annually among children under the age of five, predominantly in Southeast Asia and Africa [1]. In Vietnam, most diarrheal cases occur in children under 5 years old [2], with 71.0% of them being under 2 years old [3].

Diarrhea is classified into acute, prolonged and persistent types, depending on the duration of the condition: less than 7 days, 7–13 days, or more than 13 days, respectively [4]. Among these, acute diarrhea is the most common, but a considerable proportion (1.4–28.4%) progresses to persistent diarrhea, depending on geographical location, socioeconomic conditions and causative agents [4,5]. Persistent diarrhea has severe consequences for children’s health, significantly increasing morbidity and mortality. Although microbial infection is recognized as the main cause of both acute and persistent diarrhea, a substantial proportion of persistent cases fail to identify specific pathogens [5]. A review of 19 studies investigating the causes of persistent diarrhea in Southeast Asia and the Americas found that approximately 73% of cases were associated with multiple pathogens [6]. In Vietnam, 32.7% of acute diarrhea cases were reported to be negative for common pathogenic microorganisms [3]. Remarkably, in our study, about 44.8% of cases were classified as not-yet-identified pathogenic persistent diarrhea (NIPD) or diarrhea of unknown origin, posing a major challenge for the development of effective treatments and prevention strategies [1]. Understanding the causative pathogens of NIPD is therefore crucial for improving disease management and medical intervention.

Over the past two decades, the development of next-generation sequencing (NGS) technologies has paved the way for understanding the role of the gut microbiota in human health and disease. The gut microbiome is one of the most complex communities consisting primarily of bacteria, fungi, archaea, protozoa and viruses. The viruses compose of enveloped viruses (referred to as “viruses” in this study) and non-enveloped viruses (bacteriophages, hereafter referred to as “phages”). The distributions and diversity of these microbial communities collectively influence disease risk and overall human health [7].

In children, the gut microbiota established during the first three years of life plays a vital role in shaping a stable, balanced core microbiome in adulthood. This early development regulates not only growth but also long-term traits, behaviors and susceptibility to disease [8]. Beyond known pathogens, metagenomic analyses have revealed that gut microbiota dysbiosis is an important cause of diarrhea [9]. Numerous studies have examined viral and bacterial dysbiosis in children with diarrhea to establish a scientific basis for restoring intestinal microbial balance [10,11,12]. However, only a small number of studies have focused on the phage community, which is theoretically recognized as a key regulator of bacterial stability. Moreover, although bacterial dysbiosis in persistent diarrhea has been well documented [12], the relative abundances of viral, phage and phage-associated bacterial communities in children with NIPD have not yet been investigated.

Bacteriophages account for approximately 97.7% of the enteric viral community [13] and are closely associated with childhood diseases [13,14,15,16], including diarrhea caused by rotavirus infection [10]. However, the intestinal phage community in children with persistent diarrhea remains largely unexplored due to methodological and genetic challenges. Specifically, metagenomic investigation of the human intestinal phageome faces numerous obstacles, such as the low abundance of phages in stool, the high complexity and variability of phage genomes (including double-stranded DNA–dsDNA, single-stranded DNA–ssDNA, and single-stranded RNA–ssRNA) and their size range (typically from 4–140 kb) [13,17]. In addition, the lack of reference viral and phage’s genomes for taxonomic annotation often leads to incomplete assessments of phage compositions [18]. Differential phage enrichment and extraction methods may also produce inconsistencies in determining phageome compositions [19]. Furthermore, the natural presence of bacterial genes within phage genomes poses a significant challenge, as it can introduce errors in both phages enrichment process and downstream shotgun sequencing analyses of phage diversity [20]. To date, no studies have specifically examined the bacterial fraction as phage-associated bacterial community in phage metagenomic data. From our perspective, this genetic fraction partly reflects the bacterial populations coexisting with phages and is valuable for exploring phage–host interactions.

Based on these considerations, the present study was undertaken to (1) survey the intestinal phage composition and dysbiosis to identify viral or phage patterns potentially associated with persistent diarrhea; (2) analyze phage-associated bacterial composition and its dysbiosis; and (3) explore phage–host interactions in the gut of healthy children and children with NIPD in Vietnam using metagenomics technology. Additionally, to limit the overwhelming dominance of bacterial genes in phage metagenomic datasets, a process for phage enrichment and phage metagenome extraction was investigated.

This study, for the first time, provides novel insights into gut microbiota dysbiosis and the phageome in relation to bacterial community composition, which may be useful for developing adjunctive therapies aimed at restoring gut microbiota balance in children with NIPD.

## 2. Materials and Methods

### 2.1. Sample Collection

To investigate the gut phageome composition in children with NIPD, we collected 30 fecal specimens from healthy children and 30 fecal specimens from children with persistent diarrhea, aged between 6–24, based on the following criteria: (1) All specimens tested negative for 24 common diarrheal pathogens by real-time PCR using QIAstat-Dx Gastrointestinal Panel (Qiagen, 40724 Hilden, Germany) at Department of Molecular Biology of Infectious Diseases, Vietnam National Children’s Hospital. (2) Healthy children were defined as those without infection, diarrhea, chronic diseases or antibiotic use for at least one month prior to fecal sampling. (3) Persistent diarrheal children were defined as those experiencing an episode of diarrhea lasting at least 14 days, with signs of bacterial infections indicated by the presence of red and white blood cells under microscopic examination. The samples were collected separately following the Handbook for use of testing services (ST.XN.4.4) No. 13/CT-BVNTW issued on 22nd March 2023 by the Vietnam National Children’s Hospital, and were stored at −80 °C until further processing. The collection period extended from July 2023 to December 2024.

### 2.2. Investigation of Processes for Extracting Phage Metagenomic Material

#### 2.2.1. Investigation of Processes

The investigation of an optimal process for phage enrichment while limiting free-bacterial genes was carried out using a single fecal sample from a healthy 10-month-old boy, followed by phage metagenomic extraction using six different processes (P1–P6). Firstly, bacteria and stool substances were separated from the phage-containing supernatant as described by Nguyen et al. [21]. Briefly, the stool sample was thawed on ice, then diluted with three volumes of chilled PBS buffer (137 mM NaCl, 2.7 mM KCl, 10 mM Na_2_HPO_4_, 2 mM KH_2_PO_4_, pH 7.4), homogenized thoroughly by vortexing, and kept on ice for 10 min. The supernatant was carefully transferred into new Falcon tubes. The remaining part was repeatedly diluted with three volumes of cold PBS buffer, mixed thoroughly, then centrifuged at 500 rpm for 10 min. The collected supernatants were pooled and centrifuged at 13,000 rpm for 10 min. The resulting clarified supernatant was used for phage metagenome extraction by six processes (designated by P1–P6) (Table 1).

In processes P1 and P2, 700 μL of supernatant was directly used for phage nucleic acid extraction with either the QIAamp Viral RNA mini kit (Qiagen, 40724 Hilden, Germany) or TopPURE serum viral extraction kit (ABT, 151000, Ha Noi, Vietnam).

In processes P3-P6, the supernatant was pretreated by filtering through Millipore membranes (Merk Millipore, Burlington, MA, USA) with pore sizes of 0.45 μm (P3, P4) or 0.22 μm (P5–P6) to remove microbial cells and large particles. In processes P4-P6, after filtration, the samples were treated with DNAse I (New England Biolab, Ipswich, MA, USA) at final concentration of 1 U/mL at 37 °C for 30 min. Ethylenediaminetetraacetic acid (EDTA) was then supplemented to final concentration of 20 m), followed by incubation at 70 °C for 15 min to inactivate the DNAse I. This step aimed to eliminate free DNA present in the fecal supernatant. The processed supernatants were then subjected to phage nucleic acid extraction using the two kits described above.

#### 2.2.2. Synthesis of cDNA and Sequencing

The dsDNA, ssDNA and RNA extracted in Section 2.2.1 were used as templates to synthesize cDNAs employing HiScript II Q RT Supermix (Vazyme, Nanjing, China). The quality and quantity of the cDNAs were determined by NanoDrop NC-2000C Implen (Isogen, 3454 PW De Meern, The Netherlands). Equal amounts of cDNA were then subjected to sequencing on the Nextseq550-Illumina System (San Diego, CA, USA) at GENTIS Company (Hanoi, Vietnam).

For process P6, the obtained cDNAs fraction was combined with an equal amount of cDNAs produced in process P5 to generate sample for sequencing (Table 1). High-throughput sequencing, data quality control and annotation procedures are described below.

The similarity of viral populations obtained from processes P1-P6 were evaluated using Pearson correlation coefficients with average linkage, based on relative taxonomic abundances. Hierarchical clustering and heatmap visualization were applied by integrating phylogenetic relatedness with abundance data [22].

### 2.3. Extraction of Gut Phage Metagenomes from the Healthy and Diarrheal Children and Shotgun Metagenomic Sequencing

All 60 stool samples stored at −80 °C were thawed on ice and subjected to phageome extraction using process P6. Equal amounts of phage metagenomic material from each sample were used for cDNA synthesis with HiScript II Q RT Supermix (Vazyme, Nanjing, China). To reduce the sequencing samples for economic reasons, but without affecting the research quality, extracted cDNA samples were pooled to 20 composite samples based on similarity of bacterial diversity profiles, as determined by PCR-RFLP, RCR-DGGE and hierarchical cluster analysis of relative DNA polymorphism abundances [21]. Specifically, equal amounts of cDNAs from 30 healthy children were pooled into 10 samples (designated HV1-HV10), according to PCR-RFLP patterns of *Mbo*I-digested 16S rRNA genes [21], supported by heatmap-based similarity analysis (Figure S1). Similarly, equal amounts of cDNA from 30 NIPD cases were pooled into 10 samples (designated PV1-PV10), based on bacterial diversity analysis using PCR-DGGE of the V3 region of 16S rRNA genes [21] and heatmap-based clustering (Figure S2). The resulting 20 pooled samples (HV1-HV10, PV1-PV10) were sequenced using the Nextseq550-Illumina System (Illumina, San Diego, CA, USA) at GENTIS Company (Hanoi, Vietnam). In total, 26 metagenomic datasets—including 20 datasets pooled samples (HV1-HV10, PV1-PV10) and 6 datasets from the optimal process investigation described in Section 2.2.1—were deposited at SRA database (Table S1).

### 2.4. Taxonomic Annotation and Metagenomic Analysis

After sequencing, a quality control check was performed with the FastQC tool (version 0.11.8) (https://www.bioinformatics.babraham.ac.uk/projects/fastqc/, accessed on 8 October 2024, 2 December 2024 and 10 January 2025).The first 55 bp of each read from the DNA libraries was examined, and a Phred quality score of 30 (Q score) was applied as the benchmark for selecting high-quality reads for downstream analysis.

High-quality reads were taxonomically annotated using the Kraken2 tool with a reference database containing all bacterial, archaeal, fungal and viral genomes, applying a 90% confidence threshold [23]. Bacterial hosts were annotated by Virus-Host DB (https://www.genome.jp/virushostdb/, accessed on 13 December 2024 and 15 May 2025) [24]. Cleaned reads were then *de novo* assembled into contigs using MEGAHIT [25]. Contigs ≥ 200 bp were screened for viral sequences using VirSorter2 [26], and their completeness was estimated with CheckV [27]. Viral genomes were further classified into virulent and temperate phage types by the DeePhage tool [28,29].

### 2.5. Statistical Analysis

The taxonomic data were imported into Microsoft Excel 2010, and read numbers for each sample were normalized as percentage compositions of the total sequence volume at each taxonomic classification level. To identify significant differential abundances of gut phages or bacteria between diarrheal and healthy children, log_2_-fold-change cutoff values of ≥1.5 or ≤−1.5 were applied. Figures were generated in Microsoft Excel 2010. The reliability of observed differences was assessed using Student’s *t*-test, with statistical significance defined as *p* ≤ 0.05.

## 3. Results

### 3.1. Investigation of Optimal Processes for Phage’s Enrichment and Extraction of Phage’s Metagenome

Direct extraction of phage’s DNA/RNA from fecal supernatant without filtration and DNAse I treatment yielded 63 ng/μL with the QIAamp kit and 11 ng/μL with the TopPURE kit. Protein contamination was negligible with A260/A280 ratio > 2.0. The QIAamp kit was demonstrated as more efficient, producing higher yields both from the initial extraction after cDNA synthesis (372 ng/μL by QIAamp kit vs. 121 ng/μL).

To assess recovery efficiency, equal amounts of cDNA from processes P1–P6 were sequenced, generating 1,471,541 reads (process P3) to 2,095,218 reads (process P2). All datasets showed high quality (Q30 ≥ 92%) (Table 2), and were deposited in the SRA database (Table S1).

As summarized in Table 2, process P2 produced the highest number of classified reads (289,899 genes) but 91.77% were bacterial origin and only 7.97% were viral origin (Table 2). By contrast, the application of TopPURE kit in P1 can help to gain 21.7% reads belonging to viruses, although the kit also accumulated genes from Eukaryotes. Notably, 0.45 µm filtration in process P3 enriched viral reads 4.2-fold relative to P2, accounting for 33.6% of total classified reads by QIAamp kit. DNAse I treatment in process P4 further increased viral representation to 43.88% of classified reads (Table 2).

The TopPURE kit applied to fecal supernatant after 0.22 µm filtration and DNAse I digestion yielded the highest viral enrichment, with 48.21% of reads (Process P5). Using both extraction kits on 0.22 µm filtered and DNAse I-digested supernatant (Process P6) produced 44.8% viral reads, confirming filtration through 0.22 µm membrane and DNAse I pretreatment enhance viral metagenome recovery.

Regarding viral types, TopPURE recovered nearly tenfold more ssDNA than QIAamp (P2), whereas the QIAamp kit yielded tenfold more ssRNAs (Table 2). Although filtration and DNAse I substantially reduced both ssDNA and ssRNA, ssDNA was retained at 0.11% in process P6. Community profiling showed Caudovirales phages dominated all samples (99.9–100%) with Myoviridae as the major family, accounting for 96.87–99.25% (Table 2). The decreased abundance of common viral families Circoviridae, Genomoviridae, Coronaviridae, Caulimoviridae and Picornaviridae likely reflects removal after 0.45 μM filtration, while viruses Anelloviridae, Adenoviridae, Geminiviridae, Parvoviridae and Retroviridae persisted after 0.22 μm filtration. In particular, phage families were largely retained across P1–P6.

A total of 23 viral genera were identified from six datasets. AP22 phages were the most dominant genus (accounted for 82.6–94.4%), followed by Lactococal phage C2 (13.0% in P1, 2.0–3.1% thereafter) (Figure S3). Pearson correlation separated P3-P6 from P1–P2 (Figure S1B), highlighting the role of filtration and DNAse I pretreatment in stabilizing diversity. At the species level, *Acinetobacter* phages (strains AB3, IME200, Fri1, PD6A3 and phiAB) comprised up to 90% of viruses, alongside *Lactococcus* phages (blL67, C2) (Figure S4). Interestingly, several species were present in P1–P3 but absent in P4–P6 (Table 2), confirming efficient removal by filtration and DNAse I. Species-level clustering placed P1 apart from P2–P6 with P6 closely aligned to P4 and P5 (Figure S4B). Overall, process P6 is recommended as the optimal approach for phageome analysis of fecal samples. Combining QIAamp and TopPURE kits enables broad phage recovery, while 0.22μm filtration and DNAse I digestion are essential for phage enrichment.

### 3.2. Overview of Metagenomic Data of the Gastrointestinal Phageome and Analysis of Viral Communities in the Healthy Children and Children with NIPD

**Figure 1:**
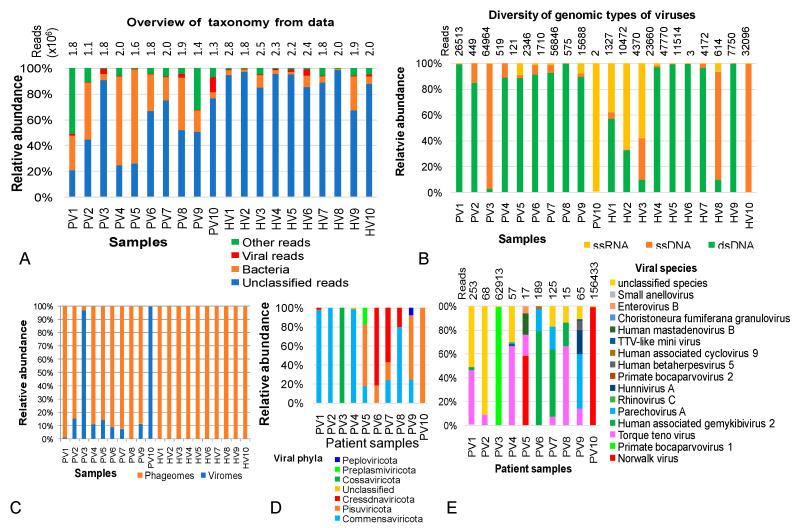
Overview picture of taxonomic annotation and viral composition in metagenomic datasets from gut phageome of heathy children and children with persistent diarrhea of unknown cause. (**A**) Taxonomic annotation at kingdom level. (**B**) Vial genomic types identified in the metagenomic data. (**C**) Relative abundances of phage and viral communities of in the samples. (**D**,**E**) Taxonomic classification and relative abundances of viruses at phylum and species levels, respectively, in samples PV1–PV10 (persistent diarrhea group) and HV1-HV10 (healthy group).

Phageomic materials from healthy and diarrheal children were prepared using process P6 and subjected to metagenomic sequencing. High-throughput sequencing generated an average of 2,200,563 reads per healthy sample (1,784,840–2,873,827 reads), and an average of 1,665,982 reads per NIPD sample (1,085,417–2,046,141 reads) with Q30>92% (Table S2). Using Kraken2, only a small proportion of reads were taxonomically classified: on average 10% in healthy samples and 47% in NIPD samples (Table S2, Figure 1A). Among classified reads, 64% of reads in healthy and 66% of reads in NIDP children belonged to bacteria, while 14% and 9.85% of reads, respectively, were viral. Notably, the proportion of classified reads varied widely among samples (Table S2).

Although dsDNA, ssDNA and ssRNA were detected across all metaegnomic datasets, their relative abundance varied by individuals (Figure 1B). Overall, dsDNA viruses dominated in most samples. Exceptions included PV10, where ssRNA viruses accounted for 99.7% of viral reads; samples PV3, HV8 and HV10, where ssDNA viruses predominated; and samples HV2 and HV3, which showed dominance of both ssDNA and ssRNA viruses (Figure 1B).

Further analysis of viral communities revealed that the viral fraction in healthy samples was exclusively composed of phages, whereas patient samples contained diverse viruses in varying proportions. Specifically, viruses were categorized into 6 phyla (including Commensaviricota, Pisuviricota, Cressdnaviricota, Cossaviricota, Preplasmiviricota and Peploviricota) (Figure 1D) and 40 species, of which 26 species belonged to *Torque teno virus* (Figure 1E, Table S4). In sample PV10, the ssRNA *Norwalk virus* (*Norovirus*) accounted for 99.98% of the viral community. In PV3, ssDNA *Primate bocaparvovirus 1* represented 99.87% of the viral fraction. *Human-associated gemykibivirus 2*, previously reported as an opportunistic pathogen, emerged as the third most abundant virus in the NIPD group, accounting for 79.3% of viral community in PV6 (Figure 1E).

### 3.3. The Intestinal Phage Compositions in the Healthy Children and Children with NIPD

**Figure 2:**
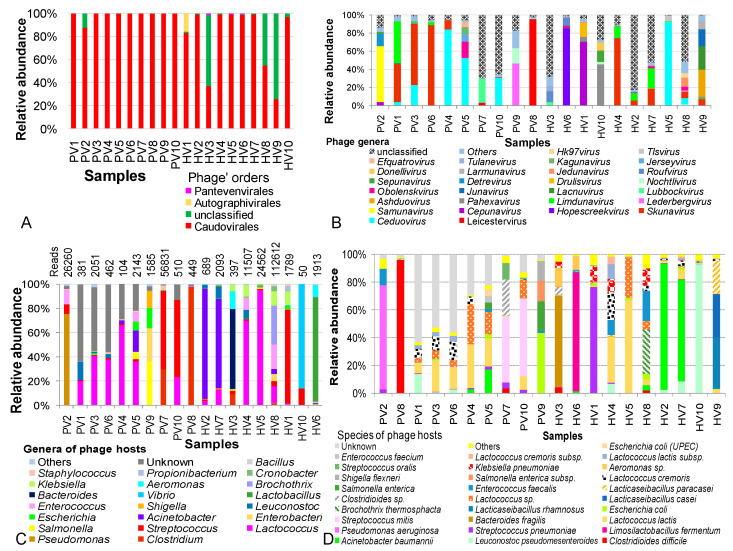
Analysis of intestinal phageome diversity in children with not-yet-identified pathogenic diarrhea and in healthy children across different taxonomic levels. (**A**) Distribution and diversity of phages at the order level. (**B**) Distribution and diversity of phages at the genus level. (**C**). Distribution and diversity of phages based on host bacteria at the genus level. (**D**) Distribution and diversity of phages based on host bacteria at the species level. PV: NIPD sample; HV: healthy sample.

Insight into phages communities in twenty samples revealed an absolute distribution of the single phage phylum Uroviricota and class Caudoviricetes with dsDNA genomes. The differences between disease and control groups were observed from the taxomomic order. In diarrheal children, Caudovirales was the overwhelmingly dominant order, occupying nearly 100% of phage communities, except in PV2, which contained 12.1% of unclassified orders. In healthy children, 8 of 10 samples were dominated by Caudovirales, however, this group appeared to be more diverse, with frequent unclassified orders, especially in HV3 and HV9, where unclassified domains accounted for 61.5 and 74.0%, respectively. Besides Caudovirales, orders of Autographivirales and Pantevenvirales appeared sparsely in four of ten heathy samples (Figure 2A). Detailed taxonomic annotation of phage communities in HV1–10 and PV1–10 metagenomic data is described in Tables S3 and S4.

At finer taxonomic levels, phage compositions varied greatly across individuals in both NIPD and controls. Siphoviridae, Myoviridae and Aliceevansviridae were consistently present, representing core families. In the NIPD, Siphoviridae, Podoviridae, Mccleskeyvirinae and Myoviridae accounted for 83.2–99.9% of phage communities, and abundant in 6/10 control samples. Compared with controls, NIPD showed increased Siphoviridae (44.2% to 80.76%, *p* = 0.011) and decreased Myoviridae (18.5% to 5.6%, *p*=0.19). The selective occurrence of Podoviridae in PV9 was proposed to be a consequence of *Rotavirus* infection in the patient donor [10]. While most NIPD samples showed no family transition, notably shifts included increased Myoviridae (82.6% in HV2, 54.7% in HV7), Salasmaviridae (64.4% in HV1), and Herelleviridae (84.0% in HV6). Similarly, Autographiviridae, Drexlerviridae, and Straboviridae were enriched in HV3–HV5, whereas unclassified families dominated in HV3 and HV8 (Table S5).

At the genus and species levels, no common phages were shared between NIPD and control samples and relative abundances were highly sample-dependent, with no consistent trends. Across groups, 98 phage genera were identified, but two NIPD and four control samples contained a substantial proportion of unclassified genera (51–83%) and more frequent in control samples (Figure 2B). *Skunavirus and Ceduovirus* were the most abundant overall, while NIPD samples showed enrichments of *Samunavirus* (61.15% in PV2), *Detrevirus* (13.65% in PV2), *Obolenskvirus* (17.31% in PV5), *Jerseyvirus* (8.65% in PV5), *Kagunavirus* (7.69% in PV5), *Leicestervirus* (95.7% in PV8), *Lederbergvirus* (46.5% in PV9) and *Nochtlivirus* (16.67% in PV9). These were either absent or negligible found in healthy samples. Conversely, *Hopescreekvirus* and *Pahexavirus* were dominant in controls but depleted in NIPD group. At the species level, 380 phage species were annotated across both groups; however, a large proportion of unclassified species was selectively present in diarrheal group, which may represent the “viral dark matter” of NIPD patients.

The consideration of bacterial host of phages (phage-infected bacteria) revealed 42 bacterial genera across groups, with an obviously greater diversity in the healthy group (Figure 2C). Unknown hosts were significantly higher in NIPD (24.5%) than the control group (*p* = 0.005). A major part of phage hosts were pathogenic genera (*Clostridium*, *Pseudomonas*, *Lactococcus*, *Streptococcus*, *Acinetobacter*, *Escherichia*, *Leuconostoc* and *Staphylococcus*). Some phage hosts were particularly dominant and exclusively detected in the NIPD group, such as *Salmonella* in PV5 (5.8%) and PV9 (36.1%), *Enterobacter* in PV9 (27.3%) and *Shigella* in PV9 (14.3%). Conversely, *Lactobacillus*, *Vibrio*, *Bacteroides*, *Brochothrix*, *Aeromonas*, *Klebsiella*, *Cronobacter*, *Propionibacterium* and *Bacillus* phages were unique to controls.

At the species level, 93 host species were totally investigated in both groups. Unknown species appeared only in the diarrhea group (24.5% vs ~ 0%, *p* = 0.05). Shared phages targeted *L. pseudomesenteroides*, *A. baumannii*, *S. pneumoniae*, *L. lactis*, *E. coli*, *Lactococcus* sp., *Clostridioides* sp., *E. faecalis*, *L. cremoris* and *S. oralis,* although their proportions varied among samples (Figure 2D). NIPD-specific phages included *C. difficile* phages (96.1% in PV8), *P. aeruginosa* phages (75.1% in PV2), *Streptococcus mitis* phages (48.3% in PV7 and 56.1% in PV10), *Salmonella enterica* phages (36.1% in PV9), *Shigella flexneri* phages (14.3% in PV9), and both *Enterococcus faecium* phages and *Escherichia coli* (UPEC) phages (in PV5). Conversely, several phage hosts were identified only in the control group, such as *Limosilactobacillus fermentum*, *Bacteroides fragilis*, *Lacticaseibacillusrhamnosus*, *Brochothrix thermosphacta*, *Lacticaseibacillus casei*, *Aeromonas* sp., *Klebsiella pneumoniae* and *Lactococcus cremoris* subsp.

### 3.4. Analysis of Bacterial Gene Origins in the Phage Metagenomic Data

Bacterial genes accounted for 66.1% and 64.2% of classified reads in NIPD and control groups, respectively (Figure 1A). At the phylum level, control samples were dominated by Firmicutes, Proteobacteria, Actinobacteria and Bacteroidetes (91.0–99.9%), with Firmicutes most dominant (31.8%). In NIPD, Actinobacteria, Bacteroidetes and Proteobacteria were remarkably depleted, resulting in higher Firmicutes (up to 55.4%) reflecting gut microbial dysbiosis associated with diarrhea.

**Figure 3:**
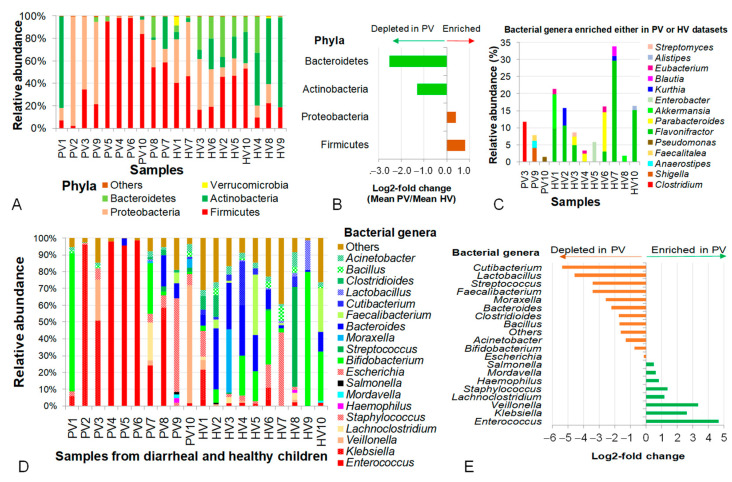
Analysis of diversity and contribution of intestinal bacteria carrying genes integrated into phageomes in children with not-yet-identified pathogenic diarrhea and healthy controls across different taxonomic levels. (**A**) Diversity and relative abundance of bacteria at phylum level. (**B**) Log2-fold changes in mean relative abundances of representative bacterial phyla. (**C**) Bacterial genera enriched in either PV or HV samples. (**D**) Distribution of bacterial genera present in both PV and HV samples. (**E**) Differentiation of bacteria at genus level expressed by log2-fold changes. PV: NIPD sample; HV: healthy sample.

As characterized by diversity patterns, the dominant distribution of Actinobacteria in PV1 was found to be similar to the pattern observed in HV4, HV8 and HV9 of the healthy group. Considering PV1 as an outlier, phylum differences were significant for Firmicutes (*p* = 0.048), Actinobacteria (*p* = 0.015) and Bacteroidetes (*p* = 0.018). Wherein, Firmicutes increased, while Bacteroidetes and Verrucomicrobia decreased in NIPD samples (Figure 3A,B), with Bacteroidetes showing the largest change (log2-fold change of −2.5) (Figure 3B).

At the genus level, bacterial genes in the phage metagenomic datasets of healthy controls were derived from a more diverse set of bacteria than in NIPD samples, including *Bifidobacterium*, *Streptococcus*, *Moraxella*, *Bacteroides*, *Faecalibacterium*, *Cutibacterium* and *Lactobacillus*. A number of genera (*Flavonifractor*, *Parabacteroides*, *Akkermansia*, *Enterobacter*, *Kurthia*, *Blautia*, *Eubacterium*, *Alistipes* and *Streptomyces*) were absent from NIPD samples but significantly enriched in the control group (Figure 3C); meanwhile, *Clostridium*, *Shigella*, *Anaerostipes*, *Faecalitalea* and *Pseudomonas* were enriched in NIPD samples (Figure 3C). In total, 20 bacterial genera were detected in both groups, though with distinct abundances. *Enterococcus* intensively appeared in both groups but was 25.4-fold higher in NIPD (*p* = 0.02), dominating in PV4 (97.9%), PV5 (95.3%) and PV6 (98.42%). *Klebsiella* was also highly abundant, accounting for 50.7%, 95.4% and 6.9% in PV3, PV2 and PV8. Other prominent genera included *Bacteroides*, *Veillonella* and *Escherichia coli* (55.7% in PV9). An abnormally high abundance of *Bifidobacterium* (82.3%) was observed in PV1. Excluding PV1, the combined average abundance of *Enterococcus*, *Klebsiella*, *Veillonella*, *Lachnoclostridium*, *Staphylococcus*, *Haemophilus*, *Mordavella*, *Salmonella* and *Escherichia* in the PV group reached 81.5%, which was 5.6-fold higher than in the HV group (*p* = 1.4 × 10^−7^) (Figure 3D,E).

**Figure 4:**
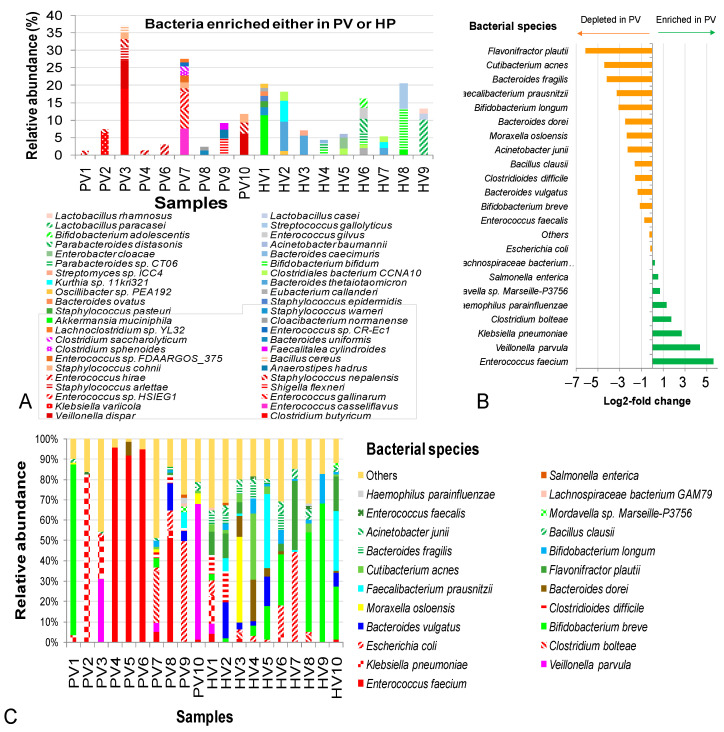
Intestinal bacteria carrying genes integrated in phageomes from children with not-yet-identified pathogenic diarrhea (NIPD) and healthy children at species level. (**A**) Bacterial species enriched in either PV samples or HV samples. (**B**) Log2-fold changes in mean bacterial abundances between PV and HV groups. (**C**) Bacterial contribution in PVs and HVs datasets. The framed species were observed only in the healthy group. PV: NIPD sample; HV: healthy sample.

At the species level, several taxa were found exclusively in healthy children, including *C. butyricum*, *V. dispar*, *E. casseliflavus*, *K. variicola*, *E. gallinarum*, *Enterococcus* sp. HSIEG1, *S. flexneri*, *S. arlettae*, *S. nepalensis*, *E. hirae*, *Anaerostipeshadrus*, *S. cohnii*, *Bacillus cereus*, *Enterococcus* sp. FDAARGOS_375, *Faecalitaleacylindroides*, *C. sphenoides*, *Bacteroides uniformis*, *C. saccharolyticum*, *Enterococcus* sp. CR-Ec1, *Lachnoclostridium* sp. YL32 and *Cloacibacterium normanense* (Figure 4A).

In contrast, some species were dominant only in diarrheal samples in a sample-dependent manner, such as *K. variicola* (6,2%) in PV2, *C. butyricum* (18.9%), *V. dispar* (8.0%), *S. arlettae* (3.8%) and *S. nepalensis* (2.4%) in PV3, and *E. casseliflavus* (7.5%), *E. gallinarum* (5.7%) and *Enterococcus* sp. HSIEG1 (4.8%) in PV7. Conversely, several probiotic species were absent in NIPD but present in control samples, including *Akkermansia muciniphila*, *S. epidermidis*, *B. ovatus*, *B. thetaiotaomicron*, *B. bifidum*, *Parabacteroides distasonis*, *B. adolescentis*, *L. paracasei*, *L. casei* and *L. rhamnosus*. Interestingly, certain pathogenic species, such as *S. warneri*, *S. pasteuri*, *S. epidermidis*, *Enterobacter cloacae*, *A. baumannii* and *S. gallolyticus* were depleted in PVs (Figure 4A).

Shared species (*S. enterica*, *Lachnospiraceae bacterium* GAM79, *E. coli* and *E. faecalis*) remained relatively stable; however, *E. coli* accumulated to 49.7% in PV9. *E. faecium* was extremely elevated in PV4 (95.8%), PV5 (91.8%), PV6 (94.7%) and PV8 (51.2%), with log2-fold change of 5.6 (48.6-fold increase). *K. pneumoniae* dominated in PV2 (82.2%) and together with *V. parvula* and *C. bolteae* were also enriched in the NIPD group, with the log2-fold changes ranging from 1.7–2.3 to 3.3–20.1 times (Figure 4B). Other pathogenic bacteria *V. parvula*, *C. bolteae*, *C. difficile* and *F. prausnitzii* were detected in the NIPD group. Conversely, beneficial taxa (*B. longum*, *F. prausnitzii*, *B. fragilis*, *Cutibacterium acnes* and *Flavonifractor plautii*) were deeply reduced in the NIPD (log2-fold changes −3.1 to −6.1 (8.6–71.0-fold decrease) (Figure 4C).

### 3.5. Primary Interaction of Bacteria and Phages Revealed by Phage Metagenomic Analysis

Based on bacterial genes in analyzed phageomes (regarded as phage-associated bacteria) and classified phages, the primary interaction of bacteria and phages in samples of NIPD and control groups was investigated.

As identified by Deephage, the number of temperate or virulent contigs in the NIPD group (1486 contigs) was found to be markedly higher than in the control group (88 contigs). The ratios of temperate to virulent phages within NIPD and control group were approximately 2:1 and 1.4:1, respectively.

**Figure 5:**
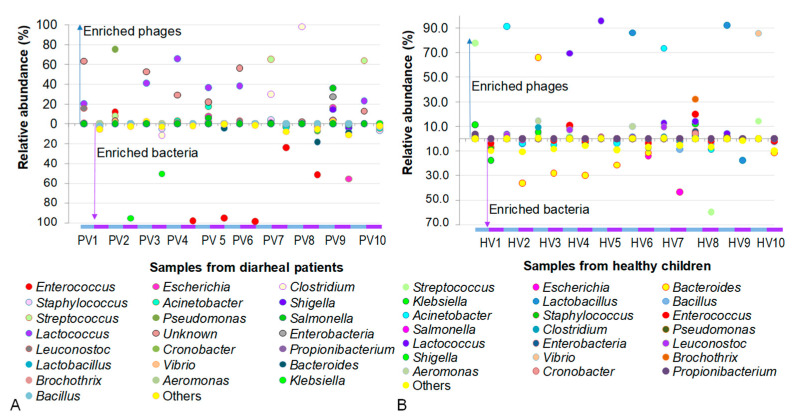
Comparison of phage and their host abundances in individual samples. (**A**) Samples from children with not-yet-identified pathogenic diarrhea. (**B**) Samples from healthy children.

A total number of 22 classified phages in PV and 21 phages in HV samples, together with their bacterial preys, were enumerated and compared across samples. In addition to highly abundant phages, some low-abundance phages were grouped as “others”, accounting for 0.4–11.3% and 1.6–10.6% of the PV and HV datasets, respectively (Figure 5).

As shown in Figure 5, an antagonistic pattern between phages and their bacterial hosts was observed in samples of NIPD group, where the presence of phages coincided with the disappearance of their corresponding host bacteria. For example, the presence of *Pseudomonas* phages in PV2 suppressed the accumulation of its host *Pseudomonas*. Conversely, the absence of *Klebsiella* phages in PV2 and PV3 was concomitant with the high abundance of *Klebsiella*. A similar trend was observed with *Enteroccoccus* host, which was enriched in PV4-PV6 and PV8 samples in the absence of its phages, as well as with *Escherichia* host, which was overwhelmed in PV9 where no corresponding phages were detected.

In datasets of healthy children, the phage–host interactions were generally observed as predator–prey dynamics, which were relatively similar to those in the PV samples. However, in some cases, phages and their bacterial hosts coexisted. For instance, *Klebsiella* and its phage co-inhabited in HV1, while *Lactobacillus* and its phage co-resided in HV9. Notably, phages appeared to regulate the bacterial populations, as reflected by the inverse relationship between their abundances. In HV1, the ratio of *Klebsiella* phages to their host was 0.7:1, whereas in HV7, the ratio of *Lactobacillus* phages to their host was 5.2:1.

## 4. Discussion

### 4.1. Filtration and DNAse I Treatment Enriched Viral Metagenome from Fecal Supernatant, and Combined Use of TopPURE and QIAamp Kits Improves Phage Recovery

The use of metagenomics technology for investigating phage communities has remained inconsistent and influenced by multiple factors. Among these, phage enrichment and phage genome extraction have been recognized as critical factors of variability in reported outcomes [30]. In this study, fecal supernatant was directly used for viral metagenome extraction. Without filtration and DNAse I treatment, the QIAamp kit yielded a higher total DNA/RNA content than the TopPURE kit; however, 91.77% of the DNA was of bacterial origin, and only 7.97% represented viral genetic materials. The filtration through a 0.45 μm membrane increased the recovery of viral metagenome to 33.62%, and subsequent DNAse I treatment further enriched it to 43.68% using the QIAamp kit. A similar enrichment pattern was observed with the TopPURE kit, where the viral fraction increased from 21.73% to 48.21% (Table 1). Interestingly, the sequencing results confirmed the effect of filtration and DNAse I treatment in removal of food-derived viruses, such as *bat-associated cyclovirus 1*, *avian coronavirus* and *cauliflower mosaic virus* (Table 1). This indicates that processing fecal supernatants with filtration and DNAse I treatment is necessary to eliminate food-derived viral contaminants. Consistent with our findings, a previous study on wastewater samples reported filtration as an effective method for concentrating viral metagenome [31], since viruses are often small in sizes and concentrated on the 0.45 μm membranes [31], whereas phages were claimed to be enriched by filtration through 0.22 μm membranes prior to viral extraction [19,32].

In our analysis, the most dominant phages were dsDNA viruses, accounting for more than 99.9% of the total viral community (Table 1). This result is in agreement with Zhang and Wang (2023), who also reported dsDNA and ssDNA phages as the dominant phages in the human gut [13]. ssDNA phages are regarded as the key players in global viromes because they are highly prevalent across diverse hosts and exhibit multiple mutations [33]. Thus the ability of TopPURE kit to increase ssDNA yield is particularly valuable.

The QIAamp^®^ Viral RNA Mini kit has been widely applied for viral metagenome extraction from various samples types, including serum [34], respiratory samples [35] and stools [36,37]. To our knowledge, however, the TopPURE serum viral extraction kit (ABT, 151000, Hanoi, Vietnam) has not previously been evaluated for stool samples. Herein, we report for the first time its successful application for viral metagenome extraction from stool samples, and show that the combination of QIAamp and TopPURE kits generates more diverse viral metagenomes.

Applying this optimized process, we extracted phage metagenomes from 60 fecal samples, which were subsequently pooled into 20 samples for sequencing. After taxonomic annotation, bacterial genes accounted for <66% of the annotated reads in both groups, while viral fractions represented an average of 14% in the control group and 9.85% in the NIPD group. For comparison, an investigation of intestinal metaviromes from 45 newborns and 45 healthy children in China, which prepared stool samples by filtering through 0.45 µm membranes, recovered only 1.0–2.0% classified reads of viral origin [38].

### 4.2. Metagenomic Data of Intestinal Phageomes in Children with Persistent Diarrhea Children Reveal Uncommon Viruses Associated with Diarrhea

The high-throughput sequencing generated an average of 2.2 million reads per healthy sample and of 1.7 million reads per NIPD sample. However, the proportion of taxonomically annotated reads in healthy samples was 4.7 times lower than in NIPD samples (Table S2). This suggests that the gut microbiome of healthy children is more diverse and harbors a greater proportion of uncharacterized or novel organisms.

Although three types of viral genomes were found in the guts of both healthy and diarrheal children, real viruses were identified only in the diarrheal children. Patient P16 (Figure S2) in PV10 was confirmed to be infected with *Norwalkvirus* (accounted for 99.98% viral communities), while patients in PV3 (including patients P07, P17) (Figure S2) were infected with *Primate bocaparvovirus* (accounted for 99.87% of the viral community). Both of these viruses are well known to cause diarrhea in humans. *Norwalkvirus* typically causes nausea, vomiting, diarrhea and stomach cramps, while *Primate bocaparvovirus* affects both respiratory and gastrointestinal systems, particularly in young children, causing cough, fever, wheezing and diarrhea [39]. Remarkably, *Human-associated gemykibivirus* 2 was detected at a high abundance (79.3%) in PV6 (including patients P13, P15, P20, P22, P23). This virus has previously been identified in children with acute encephalitis syndrome of unknown etiology in Nepal [40]. Albeit its role in diarrhea being unevidenced, *Human-associated gemykibivirus* 2 is closely related to *Gemycircularvirus*, which has been implicated in encephalitis, respiratory diseases, sepsis, pericarditis and diarrhea [41]. To our knowledge, this is the first report of *Human-associated gemykibivirus* 2 in children with persistent diarrhea of unknown etiology.

### 4.3. Variability of Intestinal Phage Compositions Between Control and the Persistent Diarrhea Groups

Compared to children with persistent diarrhea, healthy children exhibited greater diversity in gut phage orders, with a significant proportion belonging to unclassified orders. In contrast, nearly 100% of phages in the diarrhea groups were classified into order Caudovirales. Within Caudovirales, the abundance of Siphoviridae was reduced, whereas Myoviridae was increased in the healthy group. Caudoviralesis known to be expanded in inflammatory bowel disease and colitis [40], so the dominated abundance of this phage order may indicate phage dysbiosis in persistent diarrhea.

In previous studies, Microviridae, Myoviridae, Siphoviridae and unassigned families were reported as dominant in healthy children and adults [36,41]. However, in our study, Microviridae were absent. Instead, other families including Podoviridae, Mccleskeyvirinae, Salasmaviridae, Autographiviridae, Drexlerviridae and Straboviridae were abundant in specific samples. Straboviridae has been reported to be abundant in the gut of infants during the first six months of life [42], while Microviridae typically increase after 2.5 years of age [19]. Salasmaviridae are common in the respiratory tract of children at school-ages [43] and Autographiviridae and Drexlerviridae are frequently found in adults [14]. Myoviridae abundance is generally low at birth, rising to 14% of the virome at 14–23 months of age and to 21% at 28–38 months [13]. Thus, the consistently high proportion of Siphoviridae observed in the patient group suggests delayed phageome maturation in children with NIPD, most of whom were around six months old. In contrast, the shifts in viral families observed in healthy children likely reflect normal phageome maturation.

Phages detected in both healthy and diarrhea groups may represent core phages that help maintain bacterial balance in the pediatric digestive system. These included phages targeting the genera *Clostridium*, *Pseudomonas*, *Lactococcus*, *Streptococcus*, *Acinetobacter*, *Escherichia*, *Leuconostoc* and *Staphylococcus* as well as species-specific phages infecting *L. pseudomesenteroides*, *A. baumannii*, *S. pneumoniae*, *L. lactis*, *E. coli*, *Lactococcus* sp., *Clostridioides* sp., *E. faecalis*, *L. cremoris* and *S. oralis*. However, the high prevalence of *C. difficile* phages, *P. aeruginosa* phages, *S. mitis* phage, *S. enterica* phage, *S. flexneri* phages, *E. faecium phages* and *E. coli* (UPEC) phages in specific diarrhea samples suggests that these children may have experienced prior bacterial infections. Notably, phages infecting to *Enterobacter*, *Escherichia*, *Lactobacillus*, *Pseudomonas* and *Staphylococcus* were also found in high abundance in patients with type 2 diabetes or malnutrition [42].

### 4.4. Bacterial Genes in Phage Metagenomic Data Reflect Bacterial Variability and Dysbiosis in Persistent Diarrhea

Phage genomes are composed not only of phage-specific genetic material but also of bacterial genes integrated during phage packaging and maturation. Depending on the environment, bacterial genes can be abundant, and incorporated as essential components of phage genomes, accounting for 55–100% of phage metagenomic data, whereas phage genes contribute only 0–44% phage metagenomic data [20]. The bacterial genes carried by phages are diverse and encode proteins or enzymes involved in 16S rRNA, metabolism, detoxification, antibiotic resistance and biosynthesis [20,43,44]. Because this gene set reflects bacteria currently or previously present in the gut, it may serve as a useful resource for tracing past infections leading to dysbiosis at the present. However, possibly due to research purpose, to date, no studies have specifically assessed bacterial sets based on the genes incorporated in viral metagenome data.

By analyzing incorporated bacterial genes in phageomes of HV1–HV10 and PV1–PV10, we observed an increasing trend of Firmicutes and Proteobacteria and a decreasing tendency of Bacteroidetes and Verrucomicrobia in the diarrhea group in comparison to the control group. The abundances of the core bacterial phyla, including Firmicutes, Proteobacteria, Actinobacteria and Bacteroidetes, in healthy children in this study are consistent with previous reports, in which these group accounted for ~99% of bacterial communities [45,46]. The increase in Firmicutes and decrease in Bacteroidetes in diarrhea cases, leading to a dramatic decrease in the Bacteroides/ Firmicutes ratio, has also been observed in many diarrheal children infected with either viruses or bacteria [47]. In addition, the elevation of Firmicutes has been reported in children with malnutrition [48] and obesity [49]. In this study, *Bifidobacterium*, *Streptococcus*, *Moraxella*, *Bacteroides*, *Faecalibacterium*, *Cutibacterium* and *Lactobacillus* were abundant in the healthy group. Species *A. muciniphila*, *S. epidermidis*, *B. ovatus*, *B. thetaiotaomicron*, *B. bifidum*, *P. distasonis*, *B. adolescentis*, *L. paracasei*, *L. casei* and *L. rhamnosus* were highly dominant in the healthy group but depleted in the diarrhea group. Furthermore, *B. longum*, *F. prausnitzii*, *B. fragilis*, *Cutibacterium acnes* and *F. plautii* were significantly reduced in persistent diarrhea. This result indicates the severe deficiency of beneficial bacteria in the gastrointestinal system of children with persistent diarrhea of unknown etiology.

Previous studies have shown that the gut microbiota of children under 2 years old is dominated by *Bifidobacterium* before weaning, followed by *Bacteroides, Streptococcus* and also *Enterobacteriaceae* as they grow older [50]. In our study, *Bifidobacterium* was not detected in 6 diarrhea and 2 healthy samples, which may be related to (1) poor utilization of human milk oligosaccharides in diarrheal children, as described in a previous study [51]. In children fed with their mother’s breastmilk, *Bifidobacterium* can accumulate up to 80% bacterial community [51], while in healthy children fed by formular milk, the relative abundance of *Bifidobacterium* was found to be about 20% bacterial community [51], similar to the abundance of this genus in our study (19.5%); (2) disruption of bacterial dynamics caused by diarrhea. Together, these results suggest that the bacterial fraction in the metagenomic data of phageomes reflected a clear tendency of bacterial dynamics as findings from whole metagenomic DNA [51] and 16S rRNA V3-V4 amplicon analysis [47,48,49].

Some potential diarrheal pathogens were found in the persistent diarrhea that include *V. parvula*, *C. bolteae*, *C. difficile*, *F. prausnitzii* and *K. pneumoniae*, the abnormal abundance of *E. faecium* reached more than 91% of bacterial communities in PV4, PV5 and PV6 also suggest the opportunistic pathogenicity of this strain [52].

### 4.5. Bacteria–Phage Interaction Exhibits the Antagonistic and Balancing

The exploration of phage communities, concomitant with the analyzed phage-associated bacterial populations, allowed us to capture the interaction between phages and their bacterial hosts. Overall, the antagonism was reflected by the presence of phages accompanied by the absence of their host bacteria. For example, the lack of *Enterococcus* phage, *Escherichia* phage and *Acinetobacter* phage was associated with the abundance of these genera in several PV samples (PV2–PV9). Conversely, a balancing effect was observed in the coexistence of *Klebsiella* phages and *Lactobacillus* phages with their host bacteria in the healthy group. Although many probiotics, such as *Bifidobacterium*, *A. muciniphila*, *S. epidermidis*, *B. ovatus*, *B. thetaiotaomicron*, *B. bifidum*, *Parabacteroides distasonis*, *B. adolescentis*, *L. paracasei*, *L. casei* and *L. rhamnosus*, were absent in diarrheal group, we did not find specific phages of these bacteria. Remarkably, previous reports demonstrated the involvement of phages in the activity of probiotics such as *Bifidobacterium* [53].

### 4.6. Study Limitations and Future Research Directions

In this study we employed PCR–RFLP and PCR–DGGE targeting the bacterial 16S rRNA gene to cluster samples with similar microbiota profiles, pooling 30 patient samples and 30 control samples into 10 PV samples and 10 HV samples for sequencing. The pooling samples also resulted in consistent outcomes in phage, phage-associated bacteria in each group, however this approach expressed limitations.

Of the 30 persistent diarrheal children with unknown etiology, 24 children were aged 6–7 months, 3 children were 8–10 months, 2 were 11 months and 1 was 15 months. The duration of diarrhea ranged from 14 to 28 days, with 9 cases lasting 14 days, 3 lasting 15 days, 3 lasting 20 days, 5 lasting 21 days and 10 lasting 28 days. Antibiotic exposure was common, with 26 children receiving antibiotics during the acute stage prior to persistence, while 4 had not. Red and white blood cell counts in stool samples were also analyzed. Despite efforts to identify associations between phages, bacterial hosts and clinical metadata, no clear relationships were observed. Thus, phage diversity and dysbiosis in this group remained complex and have not fully explained, indicating the need for further, more detailed studies.

## 5. Conclusions

In this study, for the first time, we succeeded in the application of the TopPURE serum viral extraction kit in combination with the QIAamp Viral RNA mini kit to effectively recover diverse phage metagenomes from fecal samples of both healthy children and children with persistent diarrhea of unknown causes in Vietnam. In addition to uncommon pathogens such as *Norwalk virus*, *primate bocaparvovirus 1* and *human-associated gemykibivirus 2*, the diarrhea group was detected to be dominated by specific phages, including *Clostridioides difficile* phages, *Pseudomonas aeruginosa* phages, *Streptococcus mitis* phages, *Salmonella enterica* phages, *Shigella flexneri* phages, *Enterococcus faecium* phages and *Escherichia coli* (UPEC) phages. The bacterial genes incorporated into the phage metagenomes were mainly derived from *Escherichia*, *Acinetobacter*, *E. faecium*, *V. parvula*, *C. bolteae*, *C. difficile*, *F. prausnitzii*, *K. pneumoniae*, while genes from beneficial bacteria were remarkedly deficient. The presence of phages inhibited their host bacteria. To our knowledge, this is among the first studies to evaluate phage-associated bacteria, highlighting bacterial dysbiosis in the disease group compared to control.

## Figures and Tables

**Table 1:** Six processes for extraction of phage metagenomes from fecal supernatant.

Steps	P1	P2	P3	P4	P5	P6
Filter	No	No	0.45 µm	0.45 µm	0.22 µm	0.22 µm
Removal of free DNA	No	No	No	Yes	Yes	Yes
Metagenomic extraction kits	TopPURE	QIAamp	QIAamp	QIAamp	TopPURE	QIAamp
Synthesis of cDNA	Yes	Yes	Yes	Yes	Yes	Yes, then mixed with equal amount of cDNA from P5
Metagenomic sequencing	Yes	Yes	Yes	Yes	Yes	Yes

P1–P6: Six processes for phage’s metagenomic preparation.

**Table 2:** The metagenomic data generated from Nextseq550-Illumina system sequencer of the phageomes extracted from six processes and their taxonomic annotation.

Processes	P1		P2		P3		P4		P5		P6	
Filter	No		No		0.45 µm		0.45 µm		0.22 µm		0.22 µm	
Removal of free DNA	No		No		No		Yes		Yes		Yes	
Extracted kit	TopPURE		QIAamp		QIAamp		QIAamp		TopPURE		QIAamp+ TopPURE	
**Sequencing data**												
Q30 (%)	92		92		96		96		96		96	
Total reads	1,641,227		2,095,218		1,471,541		1,504,359		1,525,953		1,646,164	
Total classified reads	186,231		289,899		85,976		89,604		73,345		79,458	
**Diversity of kingdoms**	**Reads**	**(%)**	**Reads**	**(%)**	**Reads**	**(%)**	**Reads**	**(%)**	**Reads**	**(%)**	**Reads**	**(%)**
Bacteria	145,638	78.20	266,037	91.77	56,703	65.95	49,912	55.70	34,759	47.39	41,389	52.09
Viruses	40,469	21.73	23,100	7.97	28,903	33.62	39,314	43.88	35,359	**48.21**	35,610	**44.82**
Eukaryota	120	0.06	757	0.26	370	0.43	376	0.42	3227	4.40	2459	3.09
Archaea	4	0.00	5	0.00	0	0.00	2	0.00	0	0.00	0	0.00
**Virus types**	40,469	**(%)**	23,093	**(%)**	28,903	**(%)**	39,314	**(%)**	35,359	**(%)**	35,609	**(%)**
dsDNA	40,115	99.13	23,048	99.81	28,877	99.91	39,306	99.98	35,322	99.90	35,563	99.87
ssRNA	0	0.00	26	0.11	10	0.03	3	0.01	2	0.01	6	0.02
ssDNA	354	0.87	19	0.08	16	0.06	5	0.01	35	0.10	40	0.11
**Viral diversity at taxa**	**Numbers**											
Order	1		4		2		1		1		2	
Family	5		10		8		7		7		9	
Genus	8		14		9		7		9		12	
Species	8		13		8		6		9		12	
**Diversity at family**	**Reads**	**(%)**	**Reads**	**(%)**	**Reads**	**(%)**	**Reads**	**(%)**	**Reads**	**(%)**	**Reads**	**(%)**
Myoviridae (phage)	39193	96.87	22,811	98.75	28,685	99.25	38,973	99.13	34,770	98.33	35,123	98.64
Podoviridae (phage)	401	0.99	102	0.44	52	0.18	188	0.48	320	0.91	204	0.57
Siphoviridae (phage)	511	1.26	134	0.58	139	0.48	135	0.34	225	0.64	220	0.62
Anelloviridae (virus)	353	0.87	18	0.08	13	0.04	4	0.01	33	0.09	34	0.10
Circoviridae (virus)	1	0.002	0		0		0		0		0	
Adenoviridae (virus)	0		1	0.004	1	0.003	10	0.03	7	0.02	13	0.04
Genomoviridae (plant virus)	0		1	0.004	0		0		0		0	
Coronaviridae (virus)	0		1	0.004	0		0		0		0	
Caulimoviridae (plant virus)	0		7	0.03	0		0		0		0	
Picornaviridae (virus)	0		13	0.06	7	0.02	0		0		0	
Luteoviridae (phage)	0		12	0.05	3	0.01	3	0.01	2	0.01	6	0.02
Geminiviridae (plant virus)	0		0		3	0.01	0		2	0.01	5	0.01
Parvoviridae (phage)	0		0		0		1	0.003	0		1	0.003
Retroviridae (plant virus)	0		0		0		0		0		1	0.003

P1–P6: Six processes for phage’ enrichment and metagenomic extraction.

## Data Availability

All the metagenomic data generated from this study were deposited in SRA database with accession No: SRR34413541–SRR34413541 and SRR34424002–SRR34424013.

## Supplementary Materials

The following supporting information can be downloaded at: https://www.mdpi.com/article/10.3390/pathogens14100985/s1, Figure S1: Analysis of *Mbo*I-cleaved metagene 16S rRNA of bacteria in stool samples collected from 30 healthy children by RFLP (A), and cluster analysis of RFLP fingerprints (B); Figure S2: Analysis of V3 region in metagene 16S rRNA of bacteria in fecal samples collected from 30 persistent diarrheal children by DGGE (A), and cluster analysis of fingerprints of DGGE(B); Figure S3: Diversity (a) and Pearson correlation of genus abundances (b) of the viral genera from samples extracted by different processes; Figure S4: Diversity (A) and Pearson correlation of species abundances (B) of the viral species from samples extracted by different processes. Table S1: Detailed information of 26 metagenomic sequencing data derived from gut phageomes of healthy and unknown pathogenic persistent diarrhea children; Table S2: Overview picture on metagenomic data of gastrointestinal phageomes; Table S3: Taxonomy of bacteriophages in metagenomic data HV1-HV10; Table S4: Taxonomy of bacteriophages and viruses in metagenomic data PV1-PV10; Table S5: Relative abundance of bacteriophages at family level in metagenomic data; Table S6: Relative abundance of dominant bacteria at phyla level in metagenomic data; Table S7: Relative abundance of bacteria at genus level in metagenomic data; Table S8: Relative abundance of bacteria at species level in metagenomic data.

## Abbreviations

The following abbreviations are used in this manuscript:

NIPD	Not-yet-identified pathogenic persistent diarrhea

## References

[1] Alemu Z.A., Girmay A.M., Teklu K.T., Adugna E.A., Serte M.G., Alemayehu T.A., Likasa B.W., Collyer B., Mehari Z., Salasibew M. (2025). Prevalence of Diarrhea Disease and Associated Factors among Children under 5 Years in Geshiyaro Project Implementation Sites in Ethiopia: A Cross-Sectional Study. Health Sci. Rep..

[2] Anders K.L., Thompson C.N., Thuy N.T.V., Nguyet N.M., Tu L.T.P., Dung T.T.N., Phat V.V., Van N.T.H., Hieu N.T., Tham N.T.H. (2015). The Epidemiology and Aetiology of Diarrhoeal Disease in Infancy in Southern Vietnam: A Birth Cohort Study. Int. J. Infect. Dis..

[3] Vu N.T., Le V.P., Le H.C., Nguyen G.K., Weintraub A. (2006). Etiology and Epidemiology of Diarrhea in Children in Hanoi, Vietnam. Int. J. Infect. Dis..

[4] Moore S.R., Lima N.L., Soares A.M., Oriá R.B., Pinkerton R.C., Barrett L.J., Guerrant R.L., Lima A.A.M. (2010). Prolonged Episodes of Acute Diarrhea Reduce Growth and Increase Risk of Persistent Diarrhea in Children. Gastroenterology.

[5] de Andrade J.A.B., Fagundes-Neto U. (2011). Persistent Diarrhea: Still an Important Challenge for the Pediatrician. J. Pediatr. (Rio J.).

[6] Abba K., Sinfield R., Hart C.A., Garner P. (2009). Pathogens Associated with Persistent Diarrhoea in Children in Low and Middle Income Countries: Systematic Review. BMC Infect. Dis..

[7] Gilbert J.A., Blaser M.J., Caporaso J.G., Jansson J.K., Lynch S.V., Knight R. (2018). Current Understanding of the Human Microbiome. Nat. Med..

[8] Bankole T., Li Y. (2025). The Early-Life Gut Microbiome in Common Pediatric Diseases: Roles and Therapeutic Implications. Front. Nutr..

[9] Li Y., Xia S., Jiang X., Feng C., Gong S., Ma J., Fang Z., Yin J., Yin Y. (2021). Gut Microbiota and Diarrhea: An Updated Review. Front. Cell Infect. Microbiol..

[10] Bao S., Wang H., Li W., Wu H., Lu C., Yong L., Zhang Q., Lu X., Zhao M., Lu J. (2023). Viral Metagenomics of the Gut Virome of Diarrheal Children with Rotavirus A Infection. Gut Microbes.

[11] Gallardo P., Izquierdo M., Viver T., Bustos-Caparros E., Piras D., Vidal R.M., Harmsen H.J.M., Farfan M.J. (2024). A Metagenomic Approach to Unveil the Association between Fecal Gut Microbiota and Short-Chain Fatty Acids in Diarrhea Caused by Diarrheagenic *Escherichia coli* in Children. Microb. Cell.

[12] Tesfaw G., Siraj D.S., Abdissa A., Jakobsen R.R., Johansen Ø.H., Zangenberg M., Hanevik K., Mekonnen Z., Langeland N., Bjørang O. (2024). Gut Microbiota Patterns Associated with Duration of Diarrhea in Children under Five Years of Age in Ethiopia. Nat. Commun..

[13] Zhang Y., Wang R. (2023). The Human Gut Phageome: Composition, Development, and Alterations in Disease. Front. Microbiol..

[14] Howard A., Carroll-Portillo A., Alcock J., Lin H.C. (2024). Dietary Effects on the Gut Phageome. Int. J. Mol. Sci..

[15] Mpakosi A., Sokou R., Theodoraki M., Iacovidou N., Cholevas V., Tsantes A.G., Liakou A.I., Drogari-Apiranthitou M., Kaliouli-Antonopoulou C. (2025). The Role of Infant and Early Childhood Gut Virome in Immunity and the Triggering of Autoimmunity—A Narrative Review. Diagnostics.

[16] Zhang Y., Sharma S., Tom L., Liao Y.T., Wu V.C.H. (2023). Gut Phageome—An Insight into the Role and Impact of Gut Microbiome and Their Correlation with Mammal Health and Diseases. Microorganisms.

[17] Tobin C.A., Hill C., Shkoporov A.N. (2023). Factors Affecting Variation of the Human Gut Phageome. Annu. Rev. Microbiol..

[18] Shamash M., Maurice C.F. (2022). Phages in the Infant Gut: A Framework for Virome Development during Early Life. ISME J..

[19] Townsend E.M., Kelly L., Muscatt G., Box J.D., Hargraves N., Lilley D., Jameson E. (2021). The Human Gut Phageome: Origins and Roles in the Human Gut Microbiome. Front. Cell Infect. Microbiol..

[20] Blanco-Picazo P., Gómez-Gómez C., Tormo M., Ramos-Barbero M.D., Rodríguez-Rubio L., Muniesa M. (2022). Prevalence of Bacterial Genes in the Phage Fraction of Food Viromes. Food Res. Int..

[21] Nguyen T.Q., Dao T.K., Nguyen H.D., Phung T.B.T., Pham T.T.N., Nguyen T.V.H., Trinh T.H., Le H.C., Le T.T.H., Do T.H. (2024). Application of PCR-Based Techniques for the Identification of Genetic Fingerprint Diversity of Dominant Bacteria in Fecal Samples of Children with Diarrhea in Vietnam. Infect. Dis. Rep..

[22] Zhang Y., Huang J., Xiong Y., Zhang X., Lin Y., Liu Z. (2022). Jasmine Tea Attenuates Chronic Unpredictable Mild Stress-Induced Depressive-like Behavior in Rats via the Gut-Brain Axis. Nutrients.

[23] Wood D.E., Lu J., Langmead B. (2019). Improved Metagenomic Analysis with Kraken 2. Genome Biol..

[24] Mihara T., Nishimura Y., Shimizu Y., Nishiyama H., Yoshikawa G., Uehara H., Hingamp P., Goto S., Ogata H. (2016). Linking Virus Genomes with Host Taxonomy. Viruses.

[25] Li D., Liu C.M., Luo R., Sadakane K., Lam T.W. (2015). MEGAHIT: An Ultra-Fast Single-Node Solution for Large and Complex Metagenomics Assembly via Succinct de Bruijn Graph. Bioinformatics.

[26] Guo J., Bolduc B., Zayed A.A., Varsani A., Dominguez-Huerta G., Delmont T.O., Pratama A.A., Gazitúa M.C., Vik D., Sullivan M.B. (2021). VirSorter2: A Multi-Classifier, Expert-Guided Approach to Detect Diverse DNA and RNA Viruses. Microbiome.

[27] Nayfach S., Camargo A.P., Schulz F., Eloe-Fadrosh E., Roux S., Kyrpides N.C. (2020). CheckV Assesses the Quality and Completeness of Metagenome-Assembled Viral Genomes. Nat. Biotechnol..

[28] Wu S., Fang Z., Tan J., Li M., Wang C., Guo Q., Xu C., Jiang X., Zhu H. (2021). DeePhage: Distinguishing Virulent and Temperate Phage-Derived Sequences in Metavirome Data with a Deep Learning Approach. Giga Sci..

[29] Wu Y., Gao N., Sun C., Feng T., Liu Q., Chen W.-H. (2024). A Compendium of Ruminant Gastrointestinal Phage Genomes Revealed a Higher Proportion of Lytic Phages than in Any Other Environments. Microbiome.

[30] Shkoporov A.N., Ryan F.J., Draper L.A., Forde A., Stockdale S.R., Daly K.M., McDonnell S.A., Nolan J.A., Sutton T.D.S., Dalmasso M. (2018). Reproducible Protocols for Metagenomic Analysis of Human Faecal Phageomes. Microbiome.

[31] North D., Bibby K. (2023). Comparison of Viral Concentration Techniques for Native Fecal Indicators and Pathogens from Wastewater. Sci. Total Environ..

[32] Bikel S., Gallardo-Becerra L., Cornejo-Granados F., Ochoa-Leyva A. (2022). Protocol for the Isolation, Sequencing, and Analysis of the Gut Phageome from Human Fecal Samples. STAR Protoc..

[33] Malathi V.G., Renuka Devi P. (2019). ssDNA Viruses: Key Players in Global Virome. Virus Dis..

[34] Fanson B.G., Osmack P., Di Bisceglie A.M. (2000). A Comparison between the Phenol-Chloroform Method of RNA Extraction and the QIAamp Viral RNA Kit in the Extraction of Hepatitis C and GB Virus-C/Hepatitis G Viral RNA from Serum. J. Virol. Methods.

[35] Lista M.J., Matos P.M., Maguire T.J.A., Poulton K., Ortiz-Zapater E., Page R., Sertkaya H., Ortega-Prieto A.M., Scourfield E., O’Byrne A.M. (2021). Resilient SARS-CoV-2 Diagnostics Workflows Including Viral Heat Inactivation. PLoS ONE.

[36] Akello J.O., Bujaki E., Shaw A.G., Khurshid A., Arshad Y., Troman C., Majumdar M., O’Toole Á., Rambaut A., Alam M.M. (2023). Comparison of Eleven RNA Extraction Methods for Poliovirus Direct Molecular Detection in Stool Samples. Microbiol. Spectr..

[37] Natarajan A., Han A., Zlitni S., Brooks E.F., Vance S.E., Wolfe M., Singh U., Jagannathan P., Pinsky B.A., Boehm A. (2021). Standardized and Optimized Preservation, Extraction and Quantification Techniques for Detection of Fecal SARS-CoV-2 RNA. medRxiv.

[38] Li H., Wang H., Ju H., Lv J., Yang S., Zhang W., Lu H. (2023). Comparison of Gut Viral Communities in Children under 5 Years Old and Newborns. Virol. J..

[39] Colazo Salbetti M.B., Boggio G.A., Moreno L., Adamo M.P. (2023). Human Bocavirus Respiratory Infection: Tracing the Path from Viral Replication and Virus-Cell Interactions to Diagnostic Methods. Rev. Med. Virol..

[40] Tuladhar E.T., Shrestha S., Vernon S., Droit L., Mihindukulasuriya K.A., Tamang M., Karki L., Elong Ngono A., Jha B., Awal B.K. (2024). Gemykibivirus Detection in Acute Encephalitis Patients from Nepal. mSphere.

[41] Zucherato V.S., Giovanetti M., Costa L.O.A., Krause L.M.F., Alves D.C.C., Moreira R.M.A., Pimentel B.M.S., Haddad R., Bitencourt H.T., Ciccozzi M. (2023). Molecular Identification of the Emerging Human Gemykibivirus-2 (HuGkV-2) among Brazilian Blood Donors. Transfus. Apher. Sci..

[42] Łusiak-Szelachowska M., Weber-Dąbrowska B., Żaczek M., Borysowski J., Górski A. (2020). The Presence of Bacteriophages in the Human Body: Good, Bad or Neutral?. Microorganisms.

[43] Colomer-Lluch M., Jofre J., Muniesa M. (2011). Antibiotic Resistance Genes in the Bacteriophage DNA Fraction of Environmental Samples. PLoS ONE.

[44] Quirós P., Colomer-Lluch M., Martínez-Castillo A., Miró E., Argente M., Jofre J., Navarro F., Muniesa M. (2014). Antibiotic Resistance Genes in the Bacteriophage DNA Fraction of Human Fecal Samples. Antimicrob. Agents Chemother..

[45] Derrien M., Alvarez A.-S., de Vos W.M. (2019). The Gut Microbiota in the First Decade of Life. Trends Microbiol..

[46] Monira S., Nakamura S., Gotoh K., Izutsu K., Watanabe H., Alam N.H., Endtz H.P., Cravioto A., Ali S.I., Nakaya T. (2011). Gut Microbiota of Healthy and Malnourished Children in Bangladesh. Front. Microbiol..

[47] Xiao Q., Chen B., Zhu Z., Yang T., Tao E., Hu C., Zheng W., Tang W., Shu X., Jiang M. (2023). Alterations in the Fecal Microbiota Composition in Pediatric Acute Diarrhea: A Cross-Sectional and Comparative Study of Viral and Bacterial Enteritis. Infect. Drug Resist..

[48] Méndez-Salazar E.O., Ortiz-López M.G., Granados-Silvestre M.d.l.Á., Palacios-González B., Menjivar M. (2018). Altered Gut Microbiota and Compositional Changes in Firmicutes and Proteobacteria in Mexican Undernourished and Obese Children. Front. Microbiol..

[49] Wang J., Zhuang P., Lin B., Li H., Zheng J., Tang W., Ye W., Chen X., Zheng M. (2024). Gut Microbiota Profiling in Obese Children from Southeastern China. BMC Pediatr..

[50] Ou Y., Belzer C., Smidt H., de Weerth C. (2022). Development of the Gut Microbiota in Healthy Children in the First Ten Years of Life: Associations with Internalizing and Externalizing Behavior. Gut Microb..

[51] Casaburi G., Duar R.M., Brown H., Mitchell R.D., Kazi S., Chew S., Cagney O., Flannery R.L., Sylvester K.G., Frese S.A. (2021). Metagenomic Insights of the Infant Microbiome Community Structure and Function across Multiple Sites in the United States. Sci. Rep..

[52] Geraldes C., Tavares L., Gil S., Oliveira M. (2022). Enterococcus Virulence and Resistant Traits Associated with Its Permanence in the Hospital Environment. Antibiotics.

[53] Kiani A.K., Anpilogov K., Dautaj A., Marceddu G., Sonna W.N., Percio M., Dundar M., Beccari T., Bertelli M. (2020). Bacteriophages in Food Supplements Obtained from Natural Sources. Acta Biomed..

