# Supplementary material for: Metagenomic Analysis of the Gastrointestinal Phageome and Incorporated Dysbiosis in Children with Persistent Diarrhea of Unknown Etiology in Vietnam
Source: Pathogens. 2025 Sep 29;14(10):985. doi: 10.3390/pathogens14100985 (PMC12567195; doi:10.3390/pathogens14100985)
Supplement: Supplementary file 1 [file pathogens-14-00985-s001.zip › 5 Figures S1-S4.pptx]

## Slide 1
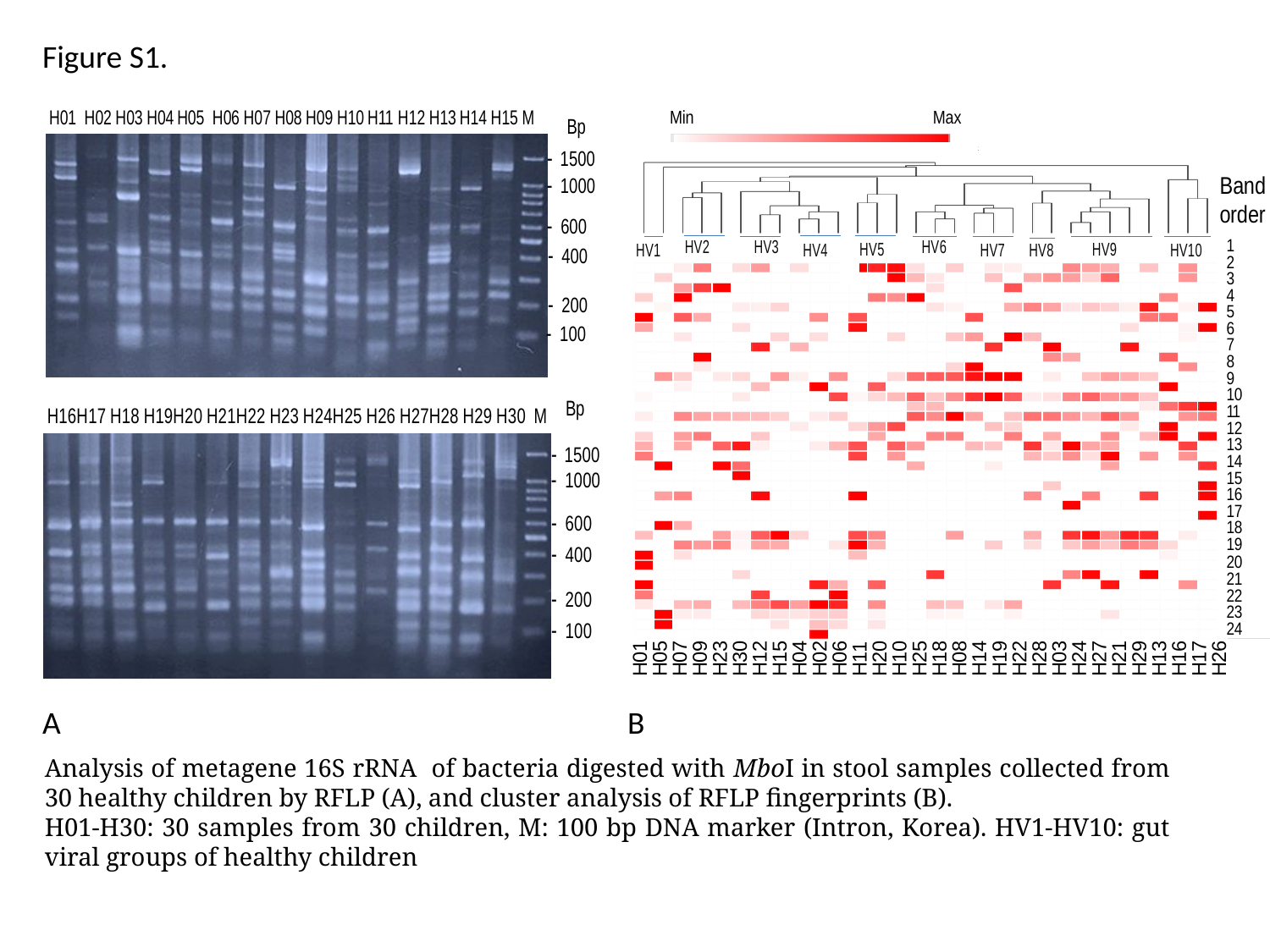

Figure S1.
A B
Analysis of metagene 16S rRNA of bacteria digested with MboI in stool samples collected from 30 healthy children by RFLP (A), and cluster analysis of RFLP fingerprints (B).
H01-H30: 30 samples from 30 children, M: 100 bp DNA marker (Intron, Korea). HV1-HV10: gut viral groups of healthy children

## Slide 2
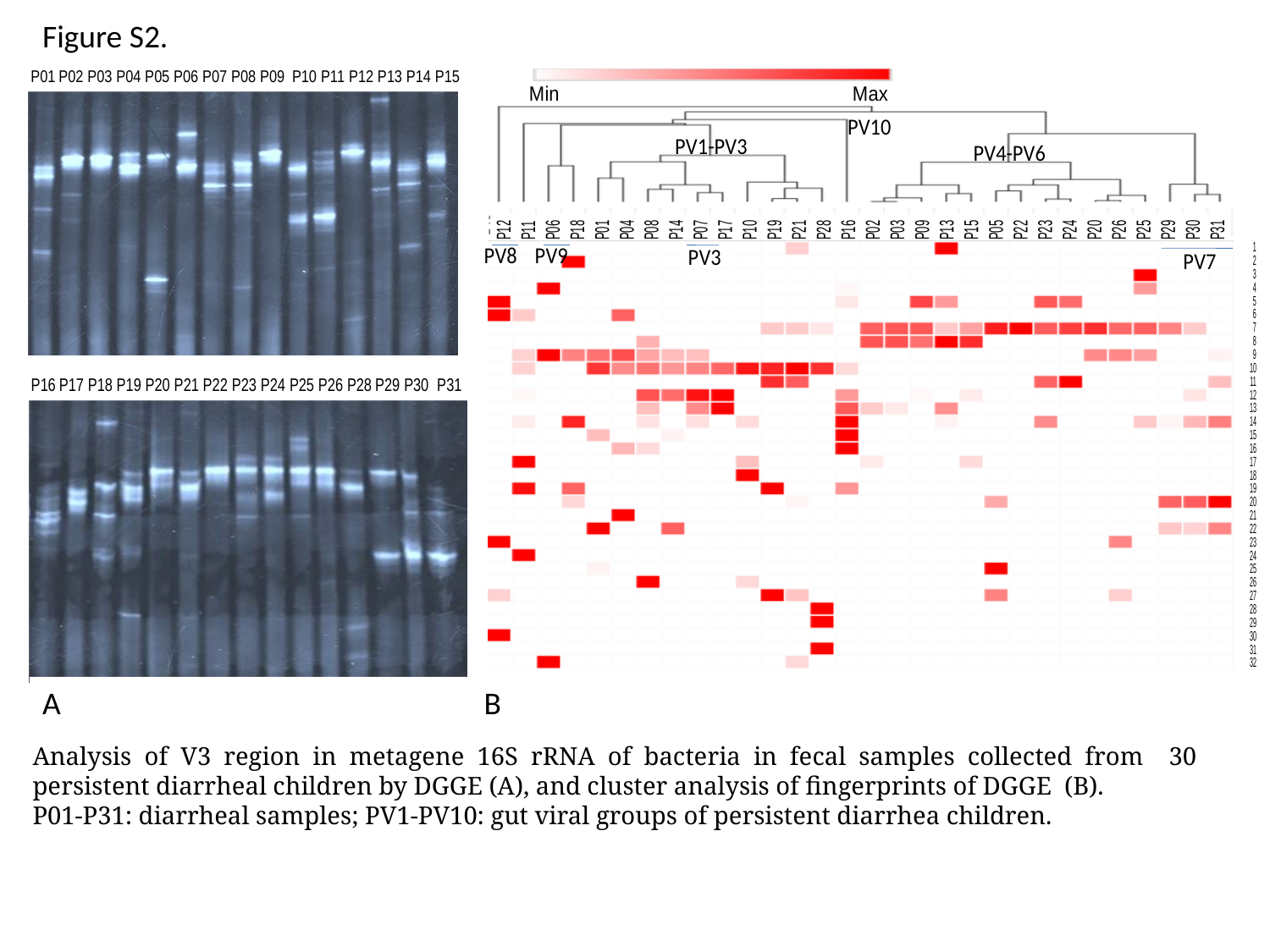

Figure S2.
A B
Analysis of V3 region in metagene 16S rRNA of bacteria in fecal samples collected from 30 persistent diarrheal children by DGGE (A), and cluster analysis of fingerprints of DGGE (B).
P01-P31: diarrheal samples; PV1-PV10: gut viral groups of persistent diarrhea children.

## Slide 3
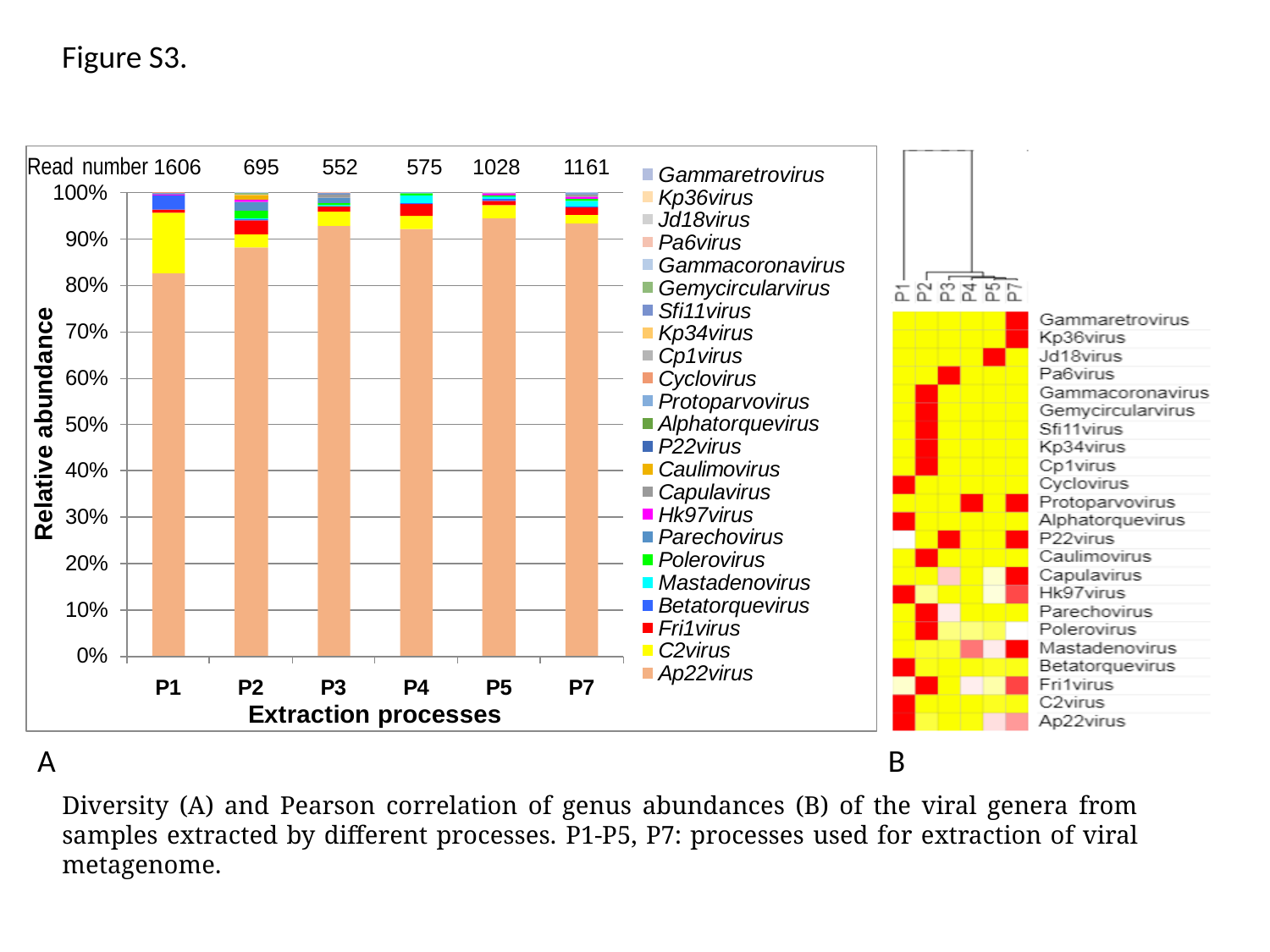

Figure S3.
A B
Diversity (A) and Pearson correlation of genus abundances (B) of the viral genera from samples extracted by different processes. P1-P5, P7: processes used for extraction of viral metagenome.

## Slide 4
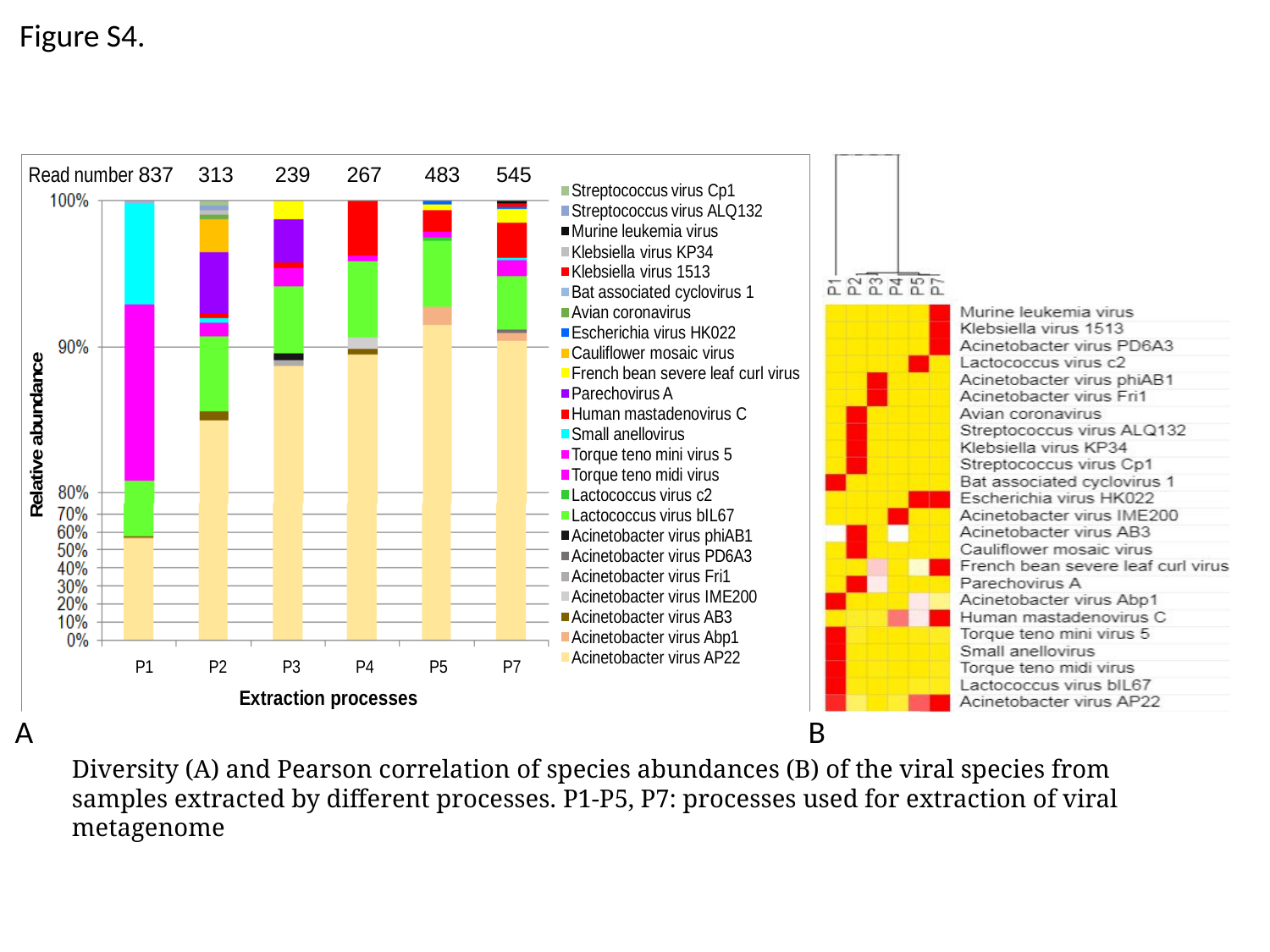

Figure S4.
A B
Diversity (A) and Pearson correlation of species abundances (B) of the viral species from samples extracted by different processes. P1-P5, P7: processes used for extraction of viral metagenome
