# Supplementary material for: Metagenomic Analysis of the Gastrointestinal Phageome and Incorporated Dysbiosis in Children with Persistent Diarrhea of Unknown Etiology in Vietnam
Source: Pathogens. 2025 Sep 29;14(10):985. doi: 10.3390/pathogens14100985 (PMC12567195; doi:10.3390/pathogens14100985)
Supplement: Supplementary file 1 [file pathogens-14-00985-s001.zip › 5 Table S1.pdf]

Table S1. Detailed information of 26 metagenomic sequencing data derived from gut phageomes of healthy and unknown pathogenic persistent diarrhea children.

Library	Group	Sampling source	Sampling date	Sample type	Description	Age	SRA Accession No.
P1	Healthy	Vietnam National Children's Hospital (VN CH)	2023	feces	Metavirome extracted directly by TopPURE Kit	Children10 months	SRR34413546
P2	Healthy	VNCH	2023	feces	Metavirome extracted directly by QIAamp kit	Children10 months	SRR34413545
P3	Healthy	VNCH	2023	feces	Metavirome extracted from 0.45 µm filtrated supernantant by QIAamp kit	Children10 months	SRR34413544
P4	Healthy	VNCH	2023	feces	Metavirome extracted from 0.45 µm filtrated and DNAseI treated supernantant by QIAamp kit	Children10 months	SRR34413543
P5	Healthy	VNCH	2023	feces	Metavirome extracted from 0.2 µm filtrated and DNAseI treated supernantant by QIAamp kit	Children10 months	SRR34413542
P6	Healthy	VNCH	2023	feces	Metavirome extracted from 0.2 µm filtrated and DNAseI treated supernantant by QIAamp kit + TopPURE kit	Children10 months	SRR34413541
PV1	Diarrhea	VNCH	2023-2024	feces	Group 1	Children (6-24 months)	SRR34424013
PV2	Diarrhea	VNCH	2023-2024	feces	Group 2	Children (6-24 months)	SRR34424012
PV3	Diarrhea	VNCH	2023-2024	feces	Group 3	Children (6-24 months)	SRR34424001
PV4	Diarrhea	VNCH	2023-2024	feces	Group 4	Children (6-24 months)	SRR34424000
PV5	Diarrhea	VNCH	2023-2024	feces	Group 5	Children (6-24 months)	SRR34423999
PV6	Diarrhea	VNCH	2023-2024	feces	Group 6	Children (6-24 months)	SRR34423998
PV7	Diarrhea	VNCH	2023-2024	feces	Group 7	Children (6-24 months)	SRR34423997

PV8	Diarrhea	VNCH	2023-2024	feces	Group 8	Children (6-24 months)	SRR34423996
PV9	Diarrhea	VNCH	2023-2024	feces	Group 9	Children (6-24 months)	SRR34423995
PV10	Diarrhea	VNCH	2023-2024	feces	Group 10	Children (6-24 months)	SRR34423994
HV1	Healthy	VNCH	2023-2024	feces	Group 1	Children (6-24 months)	SRR34424011
HV2	Healthy	VNCH	2023-2024	feces	Group 2	Children (6-24 months)	SRR34424010
HV3	Healthy	VNCH	2023-2024	feces	Group 3	Children (6-24 months)	SRR34424009
HV4	Healthy	VNCH	2023-2024	feces	Group 4	Children (6-24 months)	SRR34424008
HV5	Healthy	VNCH	2023-2024	feces	Group 5	Children (6-24 months)	SRR34424007
HV6	Healthy	VNCH	2023-2024	feces	Group 6	Children (6-24 months)	SRR34424006
HV7	Healthy	VNCH	2023-2024	feces	Group 7	Children (6-24 months)	SRR34424005
HV8	Healthy	VNCH	2023-2024	feces	Group 8	Children (6-24 months)	SRR34424004
HV9	Healthy	VNCH	2023-2024	feces	Group 9	Children (6-24 months)	SRR34424003
HV10	Healthy	VNCH	2023-2024	feces	Group 10	Children (6-24 months)	SRR34424002
HV11	Healthy	VNCH	2023-2024	feces	Metagenomic data of gut metaviromes	Children (6-11 months)	
HV12	Healthy	VNCH	2023-2024	feces	Metagenomic data of gut metaviromes	Children (12-24 months)	
HMG1	Healthy	VNCH	2023-2024	feces	Metagenomic data of gut metabacteriomes	Children (6-11 months)	
HMG1	Healthy	VNCH	2023-2024	feces	Metagenomic data of gut metabacteriomes	Children (12-24 months)	
